# Supplementary material for: Smooth Interpolating Curves with Local Control and Monotone Alternating Curvature
Source: Comput Graph Forum. 2022 Oct 6;41(5):25–38. doi: 10.1111/cgf.14600 (PMC9827861; doi:10.1111/cgf.14600)
Supplement: Supplementary file 1 — Supplement Material [file CGF-41-25-s001.zip › Local-Smooth-Interpolating-MonoCurvature/extern/clothoids/submodules/Utils/docs/api-cpp/program_listing_file_Table.cc.html]

Program Listing for File Table.cc — Utils v1.2

### Navigation

- index
- toc
- Utils »
- Program Listing for File Table.cc

# Program Listing for File Table.cc¶

↰ Return to documentation for file (`Table.cc`)

```
/*--------------------------------------------------------------------------*\
 |                                                                          |
 |  Copyright (C) 2021                                                      |
 |                                                                          |
 |         , __                 , __                                        |
 |        /|/  \               /|/  \                                       |
 |         | __/ _   ,_         | __/ _   ,_                                |
 |         |   \|/  /  |  |   | |   \|/  /  |  |   |                        |
 |         |(__/|__/   |_/ \_/|/|(__/|__/   |_/ \_/|/                       |
 |                           /|                   /|                        |
 |                           \|                   \|                        |
 |                                                                          |
 |      Enrico Bertolazzi                                                   |
 |      Dipartimento di Ingegneria Industriale                              |
 |      Universita` degli Studi di Trento                                   |
 |      Via Sommarive 9, I-38123 Povo, Trento, Italy                        |
 |      email: enrico.bertolazzi@unitn.it                                   |
 |                                                                          |
\*--------------------------------------------------------------------------*/

/*\

  Based on terminal-table:

  https://github.com/Bornageek/terminal-table

  Copyright 2015 Andreas Wilhelm

  Licensed under the Apache License, Version 2.0 (the "License");
  you may not use this file except in compliance with the License.
  You may obtain a copy of the License at

      http://www.apache.org/licenses/LICENSE-2.0

  Unless required by applicable law or agreed to in writing, software
  distributed under the License is distributed on an "AS IS" BASIS,
  WITHOUT WARRANTIES OR CONDITIONS OF ANY KIND, either express or implied.
  See the License for the specific language governing permissions and
  limitations under the License.

\*/


#ifndef DOXYGEN_SHOULD_SKIP_THIS

#include "Utils.hh"

#include <string>
#include <iostream>
#include <iomanip>
#include <sstream>
#include <cctype>
#include <algorithm>
#include <functional>
#include <stdexcept>

namespace Utils {

  namespace Table {

    using std::istringstream;
    using std::stringstream;
    using std::count;
    using std::getline;
    using std::find_if;
    using std::isspace;
    using std::setw;
    using std::left;
    using std::right;
    using std::setfill;
    using std::bind;
    using std::out_of_range;
    using std::to_string;
    using std::transform;

    Cell::Cell(
      Table*         table,
      string const & val,
      integer        colSpan
    )
    : m_Table(table)
    , m_Value(val)
    , m_ColSpan(colSpan)
    {
      m_Width = integer(val.length());
    }

    integer
    Cell::width( integer col ) const {
      integer padding    = (m_ColSpan - 1) * m_Table->cellSpacing();
      integer innerWidth = 0;

      for( integer i = 0; i < m_ColSpan; ++i )
        innerWidth += m_Table->columnWidth(col + i);

      return innerWidth + padding;
    }

    integer
    Cell::height() const {
      return integer( count( m_Value.begin(), m_Value.end(), '\n') + 1);
    }

    integer
    Cell::maxLineWidth() const {
      integer maxlen = 0;
      string  line;
      istringstream stream( m_Value );
      while( getline(stream, line) ) {
        integer len = integer( line.length() );
        if ( len > maxlen ) maxlen = len;
      }
      return maxlen;
    }

    string
    Cell::line( integer idx ) const {
      if ( idx < this->height() ) {
        istringstream stream(m_Value);
        string line;
        for( integer i = 0; i <= idx; ++i) getline(stream, line);
        this->trimLine(line);
        return line;
      }
      return "";
    }

    void
    Cell::trimLine( string & line ) const {
      auto fun = [] ( char c ) -> bool { return isspace(int(c)) == 0; };
      line.erase( line.begin(), find_if( line.begin(), line.end(), fun ) );
      line.erase( find_if( line.rbegin(), line.rend(), fun ).base(), line.end() );
    }

    string
    Cell::render( integer line, integer col ) const {
      stringstream ss;
      integer width = this->width(col);
      integer pL    = m_Table->style().paddingLeft();
      integer pR    = m_Table->style().paddingRight();

      switch( m_Align ) {
      case Alignment::LEFT:
        ss << string( pL, ' ' )
           << setw(width) << left << setfill(' ') << this->line(line)
           << string( pR, ' ' );
        break;
      case Alignment::RIGHT:
        ss << string( pL, ' ' )
           << setw(width) << right << setfill(' ') << this->line(line)
           << string( pR, ' ' );
        break;
      case Alignment::CENTER:
        {
          string  val        = this->line(line);
          integer innerWidth = width + m_Table->cellPadding();
          integer spaceLeft  = (innerWidth-integer(val.length()))/2;
          ss << string(spaceLeft, ' ')
             << setw(innerWidth - spaceLeft) << left << setfill(' ') << this->line(line);
        }
        break;
      }
      return ss.str();
    }

    Row::Row( Table * table, vecstr const & cells )
    : m_Table(table) {
      for_each(
        cells.begin(), cells.end(),
        bind(
          static_cast<void(Row::*)(string const &)>(&Row::cell),
          this,
          std::placeholders::_1
        )
      );
    }

    void
    Row::cells( vecstr const & cells ) {
      for_each(
        cells.begin(), cells.end(),
        bind(
          static_cast<void(Row::*)(string const &)>(&Row::cell),
          this,
          std::placeholders::_1
        )
      );
    }

    integer
    Row::cellWidth( integer idx ) const {
      if ( idx < this->numCells() ) return m_Cells[idx].maxLineWidth();
      return 0;
    }

    void
    Row::cellColSpan( integer idx, integer span ) {
      if ( span > 0 && idx < this->numCells() ) m_Cells[idx].colSpan(span);
    }

    void
    Row::cell( string const & value ) {
      m_Cells.push_back( Cell( m_Table, value ) );
    }

    integer
    Row::height() const {
      integer maxlen = 1;
      for_each(
        m_Cells.begin(), m_Cells.end(),
        [&maxlen]( Cell const & cell ) -> void {
          if ( cell.height() > maxlen ) maxlen = cell.height();
        }
      );
      return maxlen;
    }

    string
    Row::render() const {
      integer numColumns = m_Table->numColumns();
      integer paddingLR  = m_Table->style().paddingLeft()+
                           m_Table->style().paddingRight();
      integer numLines   = this->height();
      stringstream ss;

      Style style = m_Table->style();
      integer nc = integer(m_Cells.size());

      for ( integer l = 0; l < numLines; ++l ) {
        ss << style.borderLeft();
        for( integer c = 0; c < numColumns; ++c ) {
          if ( c < nc ) {
            Cell const & C = m_Cells[c];
            ss << C.render(l, c);
            if ( C.colSpan() > 1 ) c += C.colSpan() - 1;
          } else {
            ss << string(m_Table->columnWidth(c)+paddingLR,' ');
          }
          if ( c < numColumns-1 ) ss << style.borderMiddle();
        }
        ss << style.borderRight() << '\n';
      }
      return ss.str();
    }

    void
    Table::alignColumn( integer n, Alignment align ) {
      if ( n > this->numColumns() )
        throw out_of_range(
          "Table error: The table just has " + to_string(this->numColumns()) + " columns."
        );
      for_each(
        m_Rows.begin(), m_Rows.end(),
        [n, align]( Row & row ) -> void {
          if ( n < row.numCells() ) row[n].alignment(align);
        }
      );
    }

    void
    Table::addRow( vecstr const & row ) {
      m_Rows.push_back( Row(this, row) );
    }

    integer
    Table::cellSpacing() const {
      return this->cellPadding() + 1;
    }

    integer
    Table::cellPadding() const {
      return m_Style.paddingLeft() + m_Style.paddingRight();
    }

    typename Table::vecCell
    Table::column( integer n ) const {
      if ( n > this->numColumns() )
        throw out_of_range(
          "Table error: The table just has " + to_string(this->numColumns()) + " columns."
        );

      vecCell column(m_Rows.size());

      transform(
        m_Rows.begin(), m_Rows.end(), column.begin(),
        [n]( Row const & row ) -> Cell { return row[n]; }
      );

      return column;
    }

    integer
    Table::columnWidth( integer n ) const {
      if ( n > this->numColumns() )
        throw out_of_range(
          "Table error: The table just has " + to_string(this->numColumns()) + " columns."
        );
      integer maxlen = 0;
      auto fun = [&maxlen, n]( Row const & row ) -> void {
        if ( n < row.numCells() ) {
          integer cw = row.cellWidth(n);
          if ( cw > maxlen ) maxlen = cw;
        }
      };
      fun( m_Headings );
      for_each( m_Rows.begin(), m_Rows.end(), fun );
      return maxlen;
    }

    integer
    Table::numColumns() const {
      integer maxlen = m_Headings.numCells();
      auto fun = [&maxlen]( Row const & row ) -> void {
        integer nc = row.numCells();
        if ( nc > maxlen ) maxlen = nc;
      };
      for_each( m_Rows.begin(), m_Rows.end(), fun );
      return maxlen;
    }

    void
    Table::headings( vecstr const & headings ) {
      m_Headings = Row(this, headings);
    }

    Row &
    Table::row( integer n ) {
      if ( n >= integer(m_Rows.size()) )
        throw out_of_range(
          "Table error: The table just has " + to_string(m_Rows.size()) + " rows."
        );
      return m_Rows[n];
    }

    Row const &
    Table::row( integer n ) const {
      if ( n >= integer(m_Rows.size()) )
        throw out_of_range(
          "Table error: The table just has " + to_string(m_Rows.size()) + " rows."
        );
      return m_Rows[n];
    }

    void
    Table::rows( vecvecstr const & rows ) {
      m_Rows = vecRow();
      for_each(
        rows.begin(), rows.end(),
        [this]( vecstr const & row ) {
          m_Rows.push_back( Row(this, row) );
        }
      );
    }

    string
    Table::renderSeparator(
      char left,
      char mid,
      char right,
      char sep
    ) const {
      stringstream ss;
      ss << left;
      integer padding_LR = m_Style.paddingLeft() + m_Style.paddingRight();
      integer nc         = this->numColumns();
      for ( integer i = 0; i < nc; ++i ) {
        integer width = this->columnWidth(i) + padding_LR;
        for ( integer j = 0 ; j < width; ++j ) ss << sep;
        if ( i+1 < nc ) ss << mid;
        else            ss << right;
      }
      ss << '\n';
      return ss.str();
    }

    string
    Table::render() const {
      stringstream ss;
      string sep = this->renderSeparator(
        m_Style.borderLeftMid(),
        m_Style.borderMidMid(),
        m_Style.borderRightMid(),
        m_Style.borderMid()
      );

      if ( m_Title.length() > 0 ) {
        integer innerWidth = (this->numColumns() - 1) * this->cellSpacing() + this->cellPadding();
        for ( integer c = 0; c < this->numColumns(); ++c )
          innerWidth += this->columnWidth(c);

        integer spaceLeft = (innerWidth - integer(m_Title.length())) / 2;

        ss << m_Style.borderTopLeft()
           << string(innerWidth, m_Style.borderTop())
           << m_Style.borderTopRight()
           << '\n'
           << m_Style.borderLeft()
           << string(spaceLeft, ' ')
           << left << setw(innerWidth - spaceLeft) << setfill(' ') << m_Title
           << m_Style.borderRight()
           << '\n' << sep;
      } else {
        ss << renderSeparator(
          m_Style.borderTopLeft(),
          m_Style.borderTopMid(),
          m_Style.borderTopRight(),
          m_Style.borderTop()
        );
      }

      if ( m_Headings.numCells() > 0 )
        ss << m_Headings.render() << sep;

      if ( m_Rows.size() > 0 ) {
        for_each(
          m_Rows.begin(), --m_Rows.end(),
          [&ss, sep]( Row const & row ) -> void {
            if ( row.numCells() > 0 ) ss << row.render() << sep;
          }
        );
        ss << m_Rows.back().render();
      }

      ss << this->renderSeparator(
              m_Style.borderBottomLeft(),
              m_Style.borderBottomMid(),
              m_Style.borderBottomRight(),
              m_Style.borderBottom()
            );
      return ss.str();
    }
  }
}

#endif
```

### Quick search

### Table of Contents

- rang - Colors for your Terminal
- Zstr
- C++ API

«
hide menu

menu
sidebar
»

### Navigation

- index
- toc
- Utils »
- Program Listing for File Table.cc

© Copyright 2021, Enrico Bertolazzi.
Created using Sphinx 4.2.0.
